# The effect of long-term magnesium intake on inflammatory markers in patients with metabolic syndrome: a systematic review and meta-analysis of randomized controlled trials

**DOI:** 10.3389/fnut.2025.1692937

**Published:** 2025-10-31

**Authors:** Weinai Wang, Jiayong Wang, Yufeng Yang, Yan Shi

**Affiliations:** ^1^The First Clinical College, Liaoning University of Traditional Chinese Medicine, Shenyang, China; ^2^Department of Traditional Chinese Medicine, Liaoning Fangda General Hospital, Shenyang, China

**Keywords:** magnesium, metabolic syndrome, inflammation, meta-analysis, randomized controlled trials

## Abstract

**Background:**

Prior randomized controlled trials (RCTs) offer inconsistent evidence on extended magnesium supplementation’s impact on inflammatory markers within metabolic syndrome cases.

**Methods:**

We conducted a systematic search in Web of Science, PubMed, and Scopus, and the present meta-analysis included eight RCTs involving 444 participants. This meta-analysis adhered to PICOTS criteria and specifically evaluated the effect of oral magnesium supplementation compared to placebo on serum inflammatory markers. Only randomized controlled trials with an intervention duration of at least 2 months were included. Data were synthesized using a random-effects model, with results expressed as standardized mean differences (SMD) and 95% confidence intervals (CI). The risk of bias was assessed using the Cochrane RoB 2.0 tool.

**Results:**

Compared with placebo, magnesium intake notably lowered C-reactive protein (CRP) levels in serum (SMD = −0.327; 95% CI: −0.602 to −0.053; *p* = 0.048). Subgroup analyses revealed particularly notable improvements in serum CRP levels with oral magnesium supplementation under the following conditions: a duration of 12 weeks and 16 weeks; female sex; and administration in tablet form or capsule form. However, in other cases, this effect became non-significant (*p* > 0.05). Sensitivity analyses conducted by sequentially excluding individual studies or removing all studies with a high risk of bias showed no substantial impact on the results. No significant publication bias was observed (*p*-values for Begg’s test and Egger’s test were 0.1078 and 0.087, respectively).

**Conclusion:**

Long-term magnesium supplementation effectively improves CRP levels, with the optimal duration being 12 weeks and 16 weeks, and the preferred administration forms being tablets and capsules. The beneficial effects of long-term magnesium intake on inflammatory markers in patients with metabolic syndrome were more pronounced in women than in men.

## 1 Introduction

Metabolic syndrome, sometimes called syndrome X or insulin resistance syndrome, is deemed a critical health condition by the WHO marked by three key issues: excess belly fat, impaired insulin utilization, dyslipidemia, and hypertension. This quartet of symptoms often sets the stage for more severe complications down the road (1). Metabolic syndrome is a cluster of concurrent clinical manifestations, with diagnostic criteria typically including central obesity (increased waist circumference and visceral fat accumulation), hypertension, and elevated fasting glucose (or prediabetes/diabetes),as defined by the International Diabetes Federation (IDF) or the National Cholesterol Education Program Adult Treatment Panel III (NCEP ATP III). The prevalence of metabolic syndrome varies across regions, affecting up to one-third of adults in the United States (2). Studies reported an approximate 15.5% prevalence rate of metabolic syndrome in China in 2017 (3). The incidence of metabolic syndrome is approximately three times higher than that of diabetes, resulting in one-fourth of the global population’s occurrence roughly. This corresponds to more than 1 billion people currently affected by metabolic syndrome worldwide. A vital nutrient involved in over 300 metabolic processes, magnesium is crucial for stabilizing cardiac rhythm, reducing cardiovascular risk, facilitating energy metabolism, and exerting anti-inflammatory and antioxidant effects that may delay cellular aging. The recommended daily intake for adults ranges from 300 to 400 mg, with excellent dietary sources including dark green vegetables, nuts, whole grains, and legumes. Appropriate supplementation may optimize overall health status.

Numerous patients with metabolic syndrome exhibit elevated inflammatory marker concentrations, including C-reactive protein (CRP), tumor necrosis factor-alpha (TNF-α), and interleukin-6 (IL-6). This inflammatory response can further exacerbate insulin resistance, making the regulation of metabolic markers, such as glycemia and lipids, more difficult to control. Hence, initiating a self-perpetuating loop intensifying metabolic syndrome’s advancement (4). Contemporary research has demonstrated that regular magnesium supplementation may alleviate symptoms of metabolic syndrome by enhancing lipid profiles through various mechanisms, influencing gene expression and protein dynamics, along with the regulation of gut microbiota composition (5). Two previous studies (6, 7) have examined the effect of magnesium supplementation on inflammatory biomarkers; however, neither study restricted its participant population to individuals with specific diseases.

Consequently, this systematic review and meta-analysis comprehensively synthesizes relevant studies to assess the impact of long-term magnesium intake. In individuals diagnosed with metabolic syndrome, this study investigates how taking magnesium supplements affects the levels of specific substances in their blood that indicate inflammation. These substances include CRP, IL-6, nitric oxide (NO), malondialdehyde (MDA), total antioxidant capacity (TAC), glutathione (GSH), and TNF-α. Essentially, we are looking at whether boosting magnesium intake can dampen down inflammation in people struggling with metabolic syndrome.

## 2 Materials and methods

This review, conforming to PRISMA guidelines (8), employed a pre-registered (though unpublished) protocol, detailed in the supplement.

### 2.1 Data sources and searches

Two researchers (WW and JY) separately performed database searches, encompassing: PubMed,^1^ Scopus,^2^ and Web of Science,^3^ up to 19 June 2025, with relevant keywords. The search focused on randomized controlled trials (RCTs) examining the effects of oral magnesium versus placebo on serum inflammatory parameters. We searched these websites for terms: Metabolic Syndromes OR Syndrome, Metabolic OR Syndromes, Metabolic OR Reaven Syndrome X OR Syndrome X, Reaven OR Metabolic Syndrome X OR Insulin Resistance Syndrome X OR Metabolic Cardiovascular Syndrome OR Syndrome X, Insulin Resistance OR Metabolic X Syndrome OR Syndrome, Metabolic X OR X Syndrome, Metabolic OR Syndrome X, Metabolic OR Dysmetabolic Syndrome X OR Syndrome X, Dysmetabolic, combined with Inflammations OR Innate Inflammatory Response OR Inflammatory Response, Innate OR Innate Inflammatory Responses And magnesium.

### 2.2 Study selection

Study selection was based on the PICOTS framework:

- Population (P): adults with metabolic syndrome;
- Intervention (I): oral magnesium supplementation;
- Comparison (C): placebo control;
- Outcomes (O): serum inflammatory markers (including CRP, IL-6, TNF-α);
- Times (T): more than 2 months;
- Study design (S): randomized controlled trials (RCTs).

The exclusion criteria ruled out any research met specified disqualifying conditions that: (i) non-human studies; (ii) use of comparators other than placebo; (iii) insufficient data on serum inflammatory parameters; or (iv) magnesium supplementation duration less than 2 months. The detailed data were presented in Table 1.

**Table 1:** The meta-analysis selected studies based on the following key requirements.

PICOTS format	Inclusion criteria
Participants	Adults with metabolic syndrome
Intervention	magnesium
Comparison	control group received either placebo or untreated
Outcomes	inflammation including CRP, IL-6, TNF-α
Times	More than 2 months
Setting	Not specified
Study design	Randomized controlled trials

### 2.3 Data extraction

WW and JY, acting as separate investigators, gathered salient data from the selected publications, organizing it within a standardized Excel spreadsheet and subsequently verified item by item. For every paper, we gathered the authors’ names, publication year, country, study conditions, study design, daily magnesium dose, and follow-up span (in weeks). Additionally, we collected data on the association between magnesium or placebo and mean age, body mass index (BMI), and number of females at baseline. For studies reporting other effect sizes or using different units, all data were converted to uniform units and analyzed using effect sizes (mean ± SD).

### 2.4 Outcomes

The primary outcome was the values of serum inflammatory markers following magnesium treatment compared with placebo. The serum inflammatory markers included: CRP, IL-6 and TNF-α.

### 2.5 Quality assessment

The two researchers individually evaluated bias risk via the Cochrane Collaboration’s recommended tool (9). This tool evaluates multiple aspects of randomized controlled trial (RCT) quality, including adequacy of random sequence generation, allocation concealment, blinding of participants and personnel, blinding of outcome assessment, incomplete outcome data (e.g., attrition evaluation), selective outcome reporting, and other potential sources of bias. According to Cochrane Handbook recommendations, potential ratings included low risk of bias, high risk, or unclear (10).

### 2.6 Data synthesis and analysis

STATA 14.0 (STATA Corp) was used for all data analyses. In the study, the mean changes in inflammatory cytokines and their standard deviations between baseline and study endpoint across the intervention and comparator arms were used to assess the overall effect size. When mean and standard deviation were absent from source material, we employed this computation: Additionally, the researchers transformed SEs, 95% CIs, and IQRs to standard deviation. In the presence of heterogeneity, we calculated pooled effect sizes using a random-effects model (11). Otherwise, a fixed-effects model was employed. Study variation was assessed via Cochran’s Q and *I*^2^ statistics; *p* < 0.05 and *I*^2^ > 50% suggested noteworthy clinical heterogeneity (12). To identify sources of heterogeneity, subgroup analyses were conducted based on sex, type of magnesium and intervention duration.

Funnel plot symmetry, along with Kendall’s tau (13) and Egger’s test, gauged publication bias (14). Significance was set at *p* < 0.05 across all analyses.

## 3 Results

### 3.1 Search results

**Figure 1:**
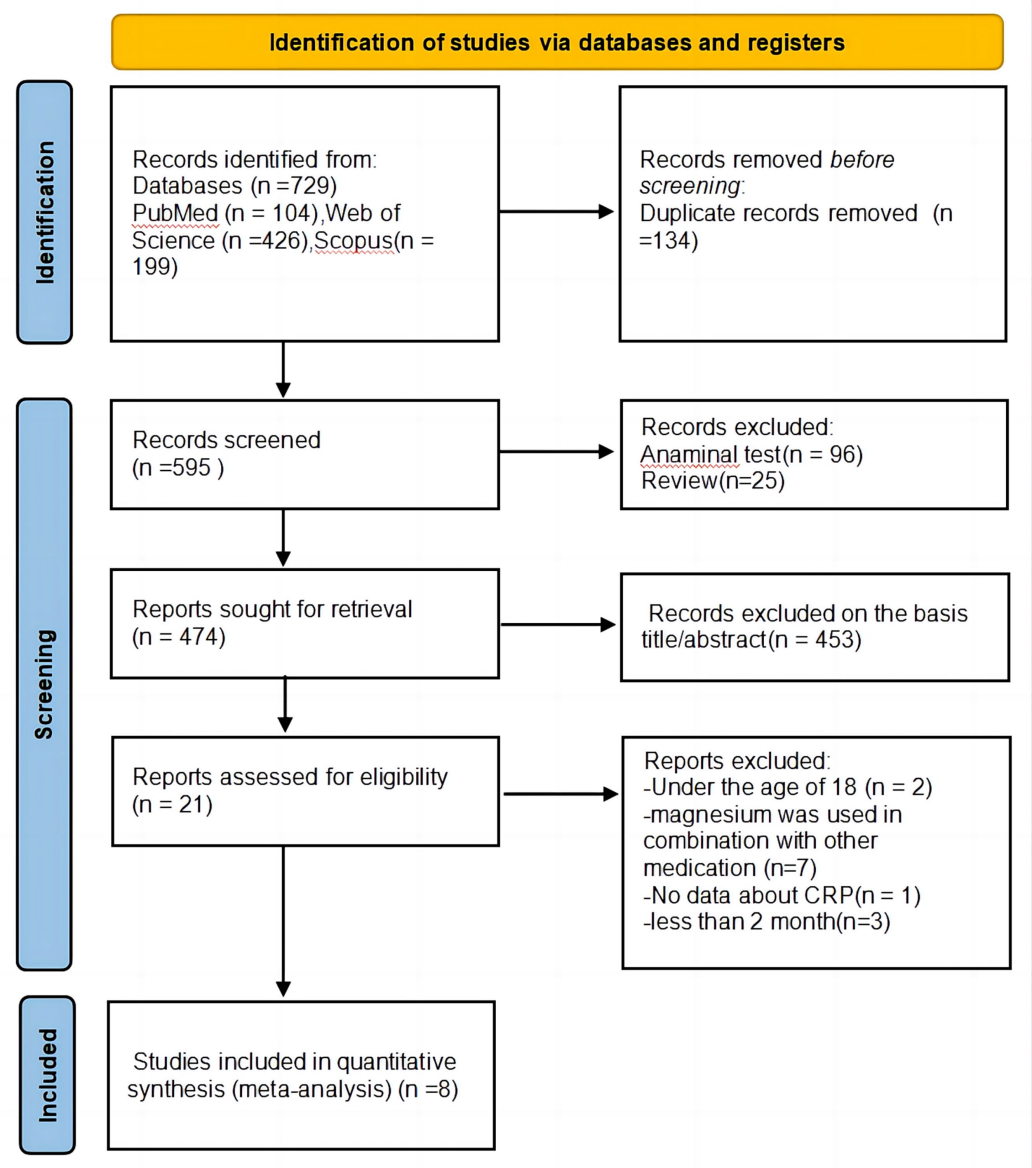
Flow diagram for identifying eligible studies.

Figure 1 presents the PRISMA flow diagram of literature search. Our initial search identified 729 articles from three databases and alternative origins. Duplicate removal (*n* = 134) left 595 records. After reviewing titles and abstracts, 453 studies were deemed unrelated to the subject. The remaining 21 full-text articles were thoroughly assessed, resulting in the exclusion of 13 studies for the following reasons: two studies involved participants under 18 years old; seven studies examined magnesium in combination with other medications; only one study did not report CRP data; and three studies had an intervention duration shorter than 2 months. Consequently, a total of eight studies (15–22) satisfied prerequisites for, and were incorporated into, this meta-analysis.

### 3.2 Study characteristics

This systematic review and meta-analysis encompassed eight studies with a total of 444 participants. The studies were published between 2010 and 2025, with treatment group sample sizes ranging from 11 (15) to 40 (21), and participant ages from 18 years and above. Among the included studies, three were conducted in Iran (19–21), three in Mexico (15, 17, 22), one in Netherlands (18), and one in Brazil (16). Of these trials, two enrolled only female participants (16, 22) while six included both sexes (15, 17–21). Participants’ average age varied between 40.44 (17) and 62 years (18). The mean BMI ranged from 26.65 (20) to 36.85 (22) kg/m^2^. Three studies included participants with diabetes and its complications (19–21), two studies involved prediabetic patients (15, 17), and two other studies enrolled obese and overweight participants (18, 22). Additionally, one trial (16) included participants meeting metabolic syndrome diagnostic criteria. The administered forms of oral magnesium included tablets (16, 19–21), capsules (18), and solution (15, 17, 22). Magnesium types comprised chelated magnesium (16) and elemental magnesium (15, 17–22). The daily magnesium dosage ranged from 150 mg (lowest dose, 18) to 450 mg (highest dose, 21). The intervention duration varied from 12 weeks (shortest duration, (15–17, 19, 21)) to 24 weeks (longest duration, (18, 20)). Detailed characteristics of eligible trials are presented in the Table 2.

**Table 2:** Characteristics of eligible studies on the effects of magnesium supplementation on inflammatory biomarkers.

First author (year)	Country	Condition	Sex	Sample size (magnesium /placebo)	Mean age (year)	Mean BMI (kg/m^2^)	RCT design	Duration (week)	Type of magnesium	Intervention	
										Treatment group	Control group
Lima de Souza et al. (16)	Brasil	Metabolic syndrome	Female	72 (35/37)	45.62	35.29	Parallel (double-blinded)	12	Tablet	400 mg/d, chelated Mg	Placebo
Joris et al. (18)	The Netherlands	Overweight/obesity	both	51 (26/25)	62	29.6	Parallel (double-blinded)	24	Capsule	350 mg/d, elemental Mg	Placebo (starch)
Razzaghi et al. (19)	Iran	Diabetic foot ulcer	both	70 (35/35)	59.55	27.2	Parallel (double-blinded)	12	Tablet	150 mg/d, elemental Mg	Placebo
Talari et al. (20)	Iran	Diabetic hemodialysis	both	54 (27/27)	60.3	26.65	Parallel (double-blinded)	24	Tablet	250 mg/d, elemental Mg	Placebo
Sadeghian et al. (21)	Iran	DM and early stage nephropathy	both	80 (40/40)	42	31.05	Parallel (double-blinded)	12	Tablet	250 mg/d, elemental Mg	Placebo
Rodriguez-Hernandez et al. (22)	Mexico	obesity	Female	38 (20/18)	48	36.85	Parallel	16	Solution	450 mg/d, elemental Mg	No placebo
Simental-Mendia et al. (15)	Mexico	Prediabetes	both	22 (11/11)	42.9	31.8	Parallel (double-blinded)	12	Solution	382 mg/d, elemental Mg	Placebo
Simental-Mendia et al. (17)	Mexico	Prediabetes	both	57 (29/28)	40.44	30.25	Parallel (double-blinded)	12	Solution	382 mg/d, elemental Mg	Placebo

### 3.3 Quality assessment

The quality assessment conducted according to Cochrane methodology revealed that two studies (23) showed moderate bias risk, while the remaining six included trials demonstrated low bias risk. The detailed results across all domains are presented in Table 3.

**Table 3:** Cochrane risk of bias assessment.

Study	Random sequence generation	Allocation concealment	Selective reporting	Other sources of bias	Blinding (participants and personnel)	Blinding (outcome assessment)	Incomplete outcome data	General risk of bias
Lima de Souza et al. (16)	L	U	H	H	L	U	L	Moderate
Joris et al. (18)	L	U	L	L	L	U	L	Low
Razzaghi et al. (19)	L	U	L	L	L	L	L	Low
Talari et al. (20)	L	U	H	L	L	L	L	Low
Sadeghian et al. (21)	L	U	H	L	L	L	L	Low
Rodriguez-Hernandez et al. (22)	L	H	L	U	U	U	L	Low
Simental-Mendia et al. (15)	L	U	L	H	L	U	L	Low
Simental-Mendia et al. (17)	L	U	H	H	L	U	L	Moderate

L, low risk of bias; H, high risk of bias; U, unclear risk of bias. General low risk of bias: <2 high risk. General moderate risk of bias: =2 high risk. General bad: >2 high risk.

### 3.4 Meta-analysis

**Figure 2:**
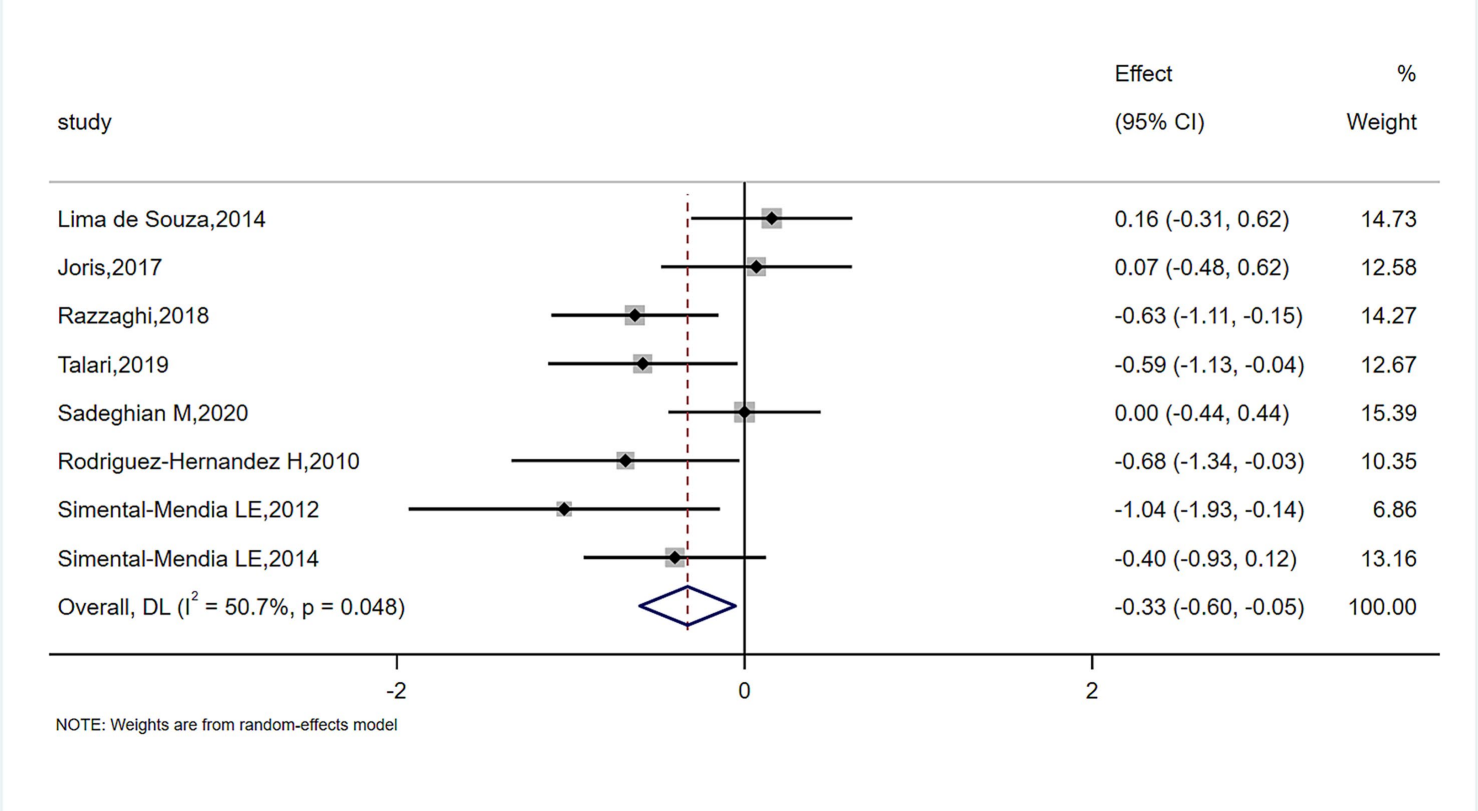
Forrest plot of the effect of magnesium versus placebo on serum C-reactive protein.

**Figure 3:**
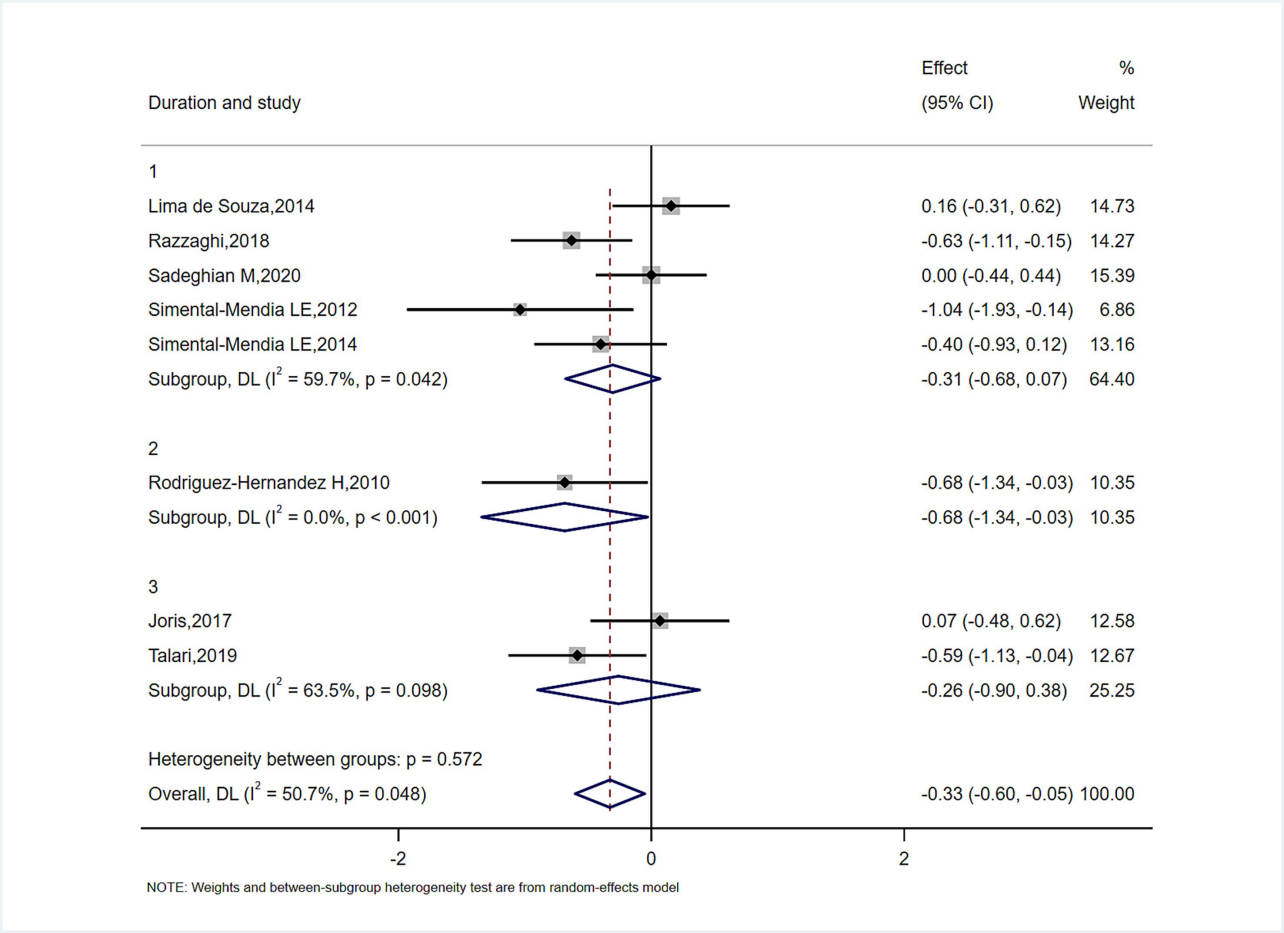
Subgroup analysis for the effect of magnesium on serum CRP in duration.

The meta-analysis of magnesium’s impact versus placebo biomarkers is showcased in Table 4. Among 444 participants from eight randomized controlled trials (RCTs), magnesium intake demonstrably lowered blood CRP concentration relative to the control group (SMD = −0.327; 95% CI: −0.602 to −0.053; *p* = 0.048), despite observed substantial heterogeneity (*I*^2^ = 50.7%) (Figure 2). For inflammatory parameters with fewer than 3 RCTs, magnesium supplementation showed no significant effects on serum IL-6 or tumor necrosis factor-alpha levels (Figure 3).

**Table 4:** Meta-analysis of magnesium supplementation on serum inflammatory parameters.

Inflammatory parameter	Number of comparisons	Number of participants	SMD	95%CI		*p*-value	*I*^2^ (%)
CRP	8	444	−0.327	−0.602	−0.053	0.048	50.7
IL-6	2	37	−0.36	−0.826	−0.106	0.186	43
TNF-α	2	37	0.08	−0.38	0.54	0.473	0

### 3.5 Subgroup analysis

**Figure 4:**
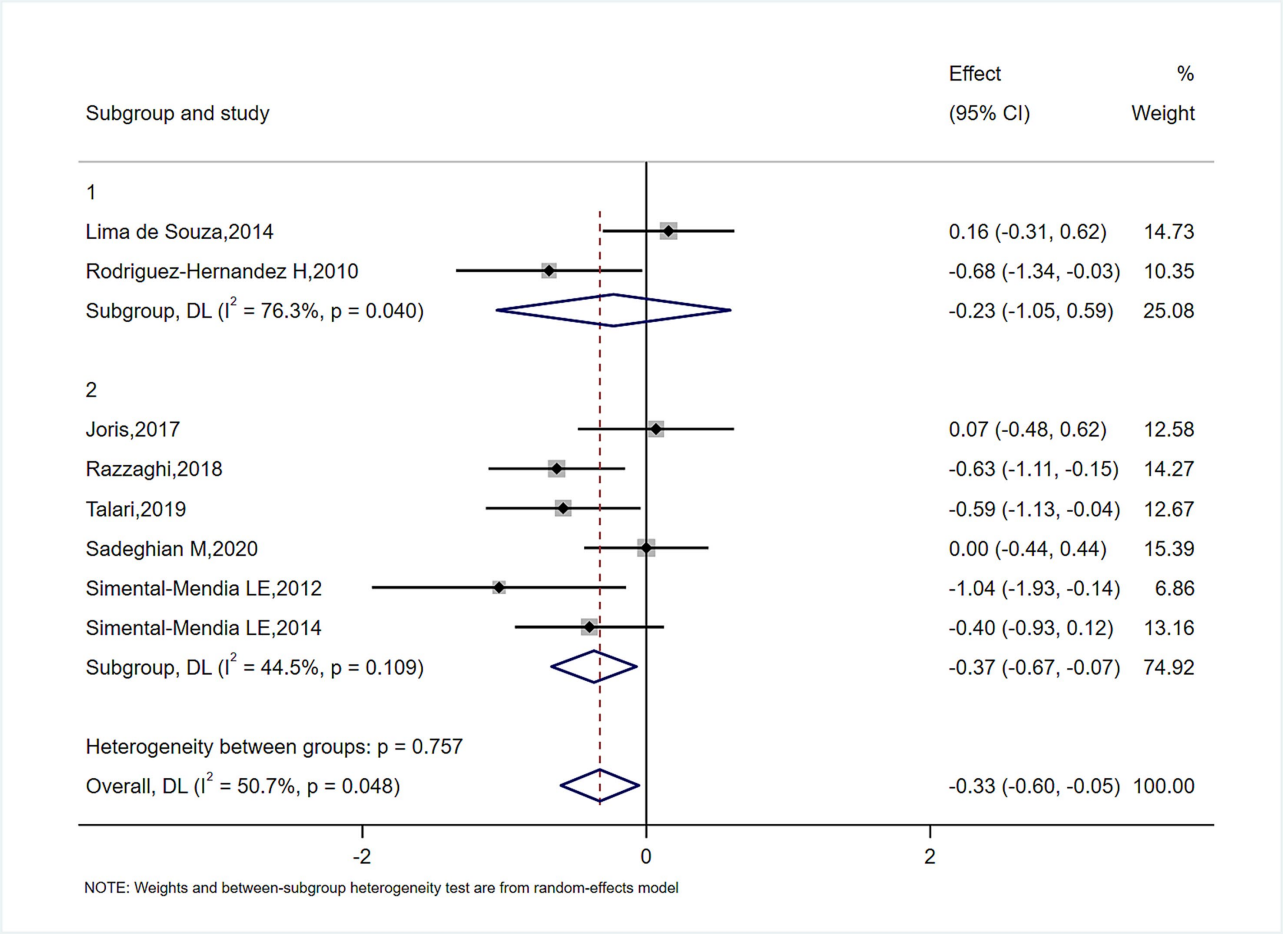
Subgroup analysis for the effect of magnesium on serum CRP in sex.

**Figure 5:**
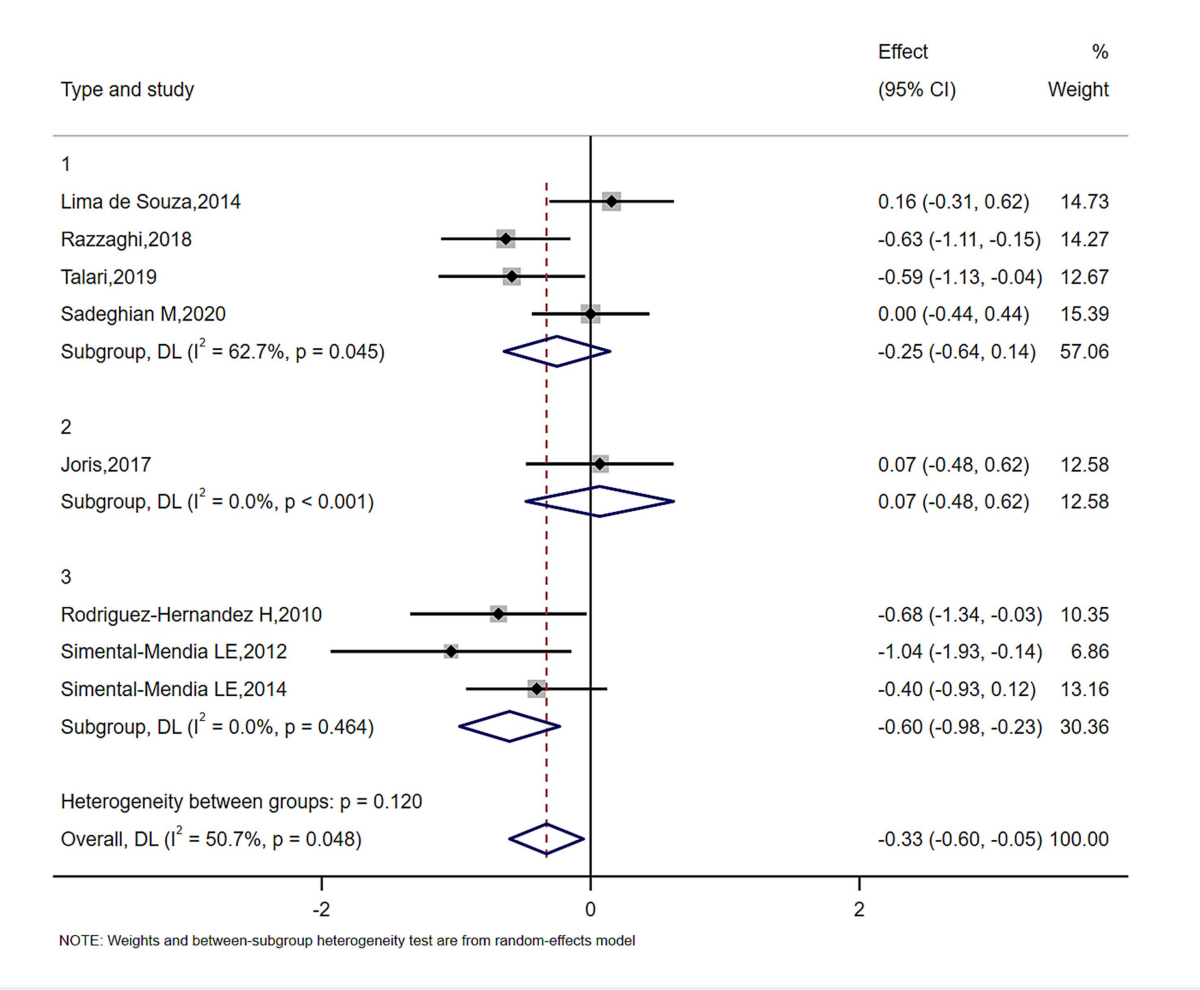
Subgroup analysis for the effect of magnesium on serum CRP in type of magnesium.

**Figure 6:**
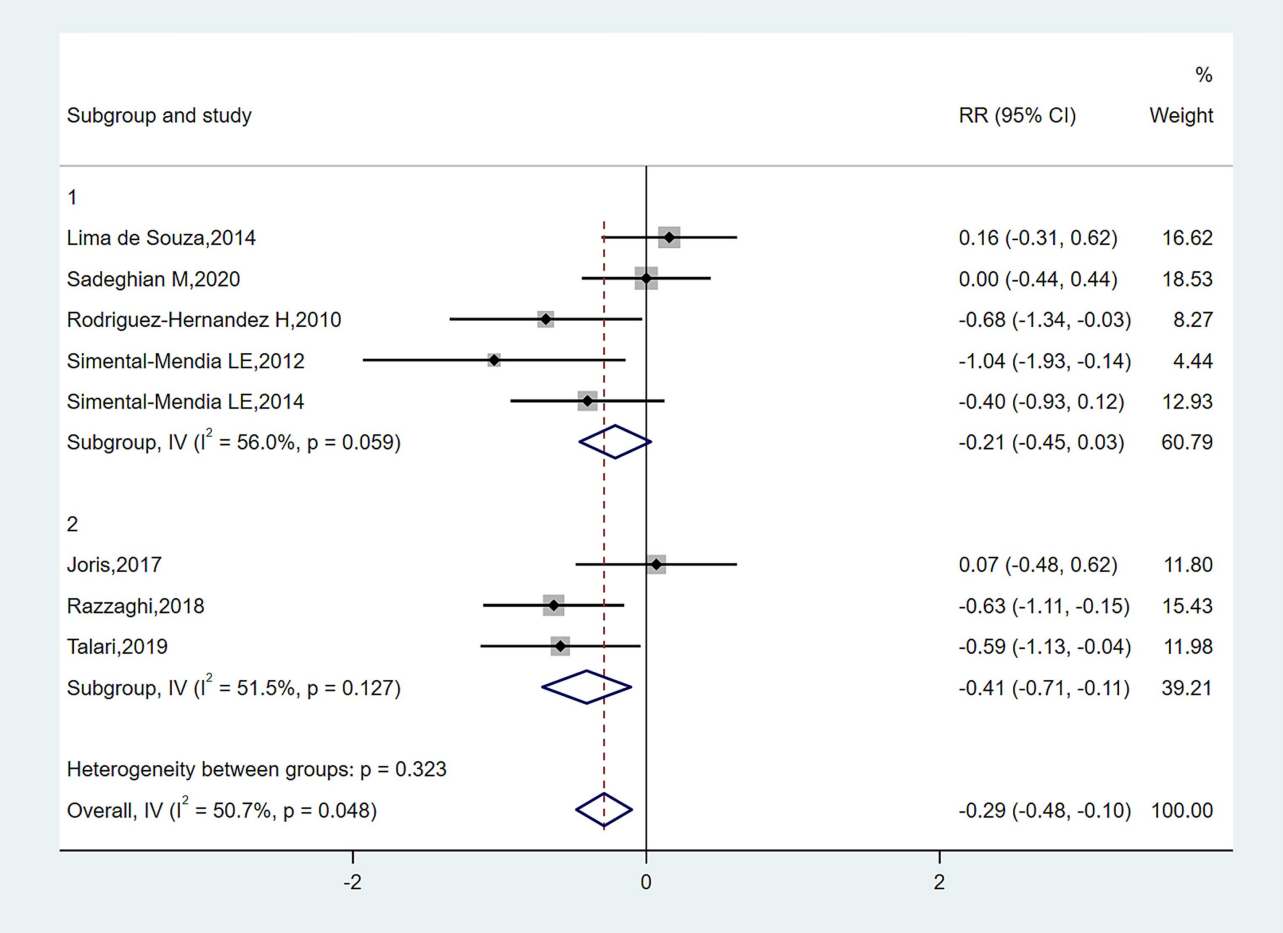
Subgroup analysis for the effect of magnesium on serum CRP in age.

In subgroup analyses of Figures 4–6, the significant improvement in serum CRP levels with oral magnesium supplementation was particularly evident under the following conditions: duration of 12 weeks (SMD, −0.31; 95% CI, −0.68 to 0.07; *p* = 0.042) and 16 weeks (SMD, −0.68; 95% CI, −1.34 to −0.63; *p* = 0.000); female sex (SMD, −0.23; 95% CI, −1.05 to 0.59; *p* = 0.040); and administration forms of tablets (SMD, −0.25; 95% CI, −0.64 to 0.14; *p* = 0.045) and capsules (SMD, −0.07; 95% CI, −0.48 to 0.62; *p* = 0.000). However, this effect became non-significant in other subgroups (*p* > 0.05).

### 3.6 Sensitivity analysis

**Figure 7:**
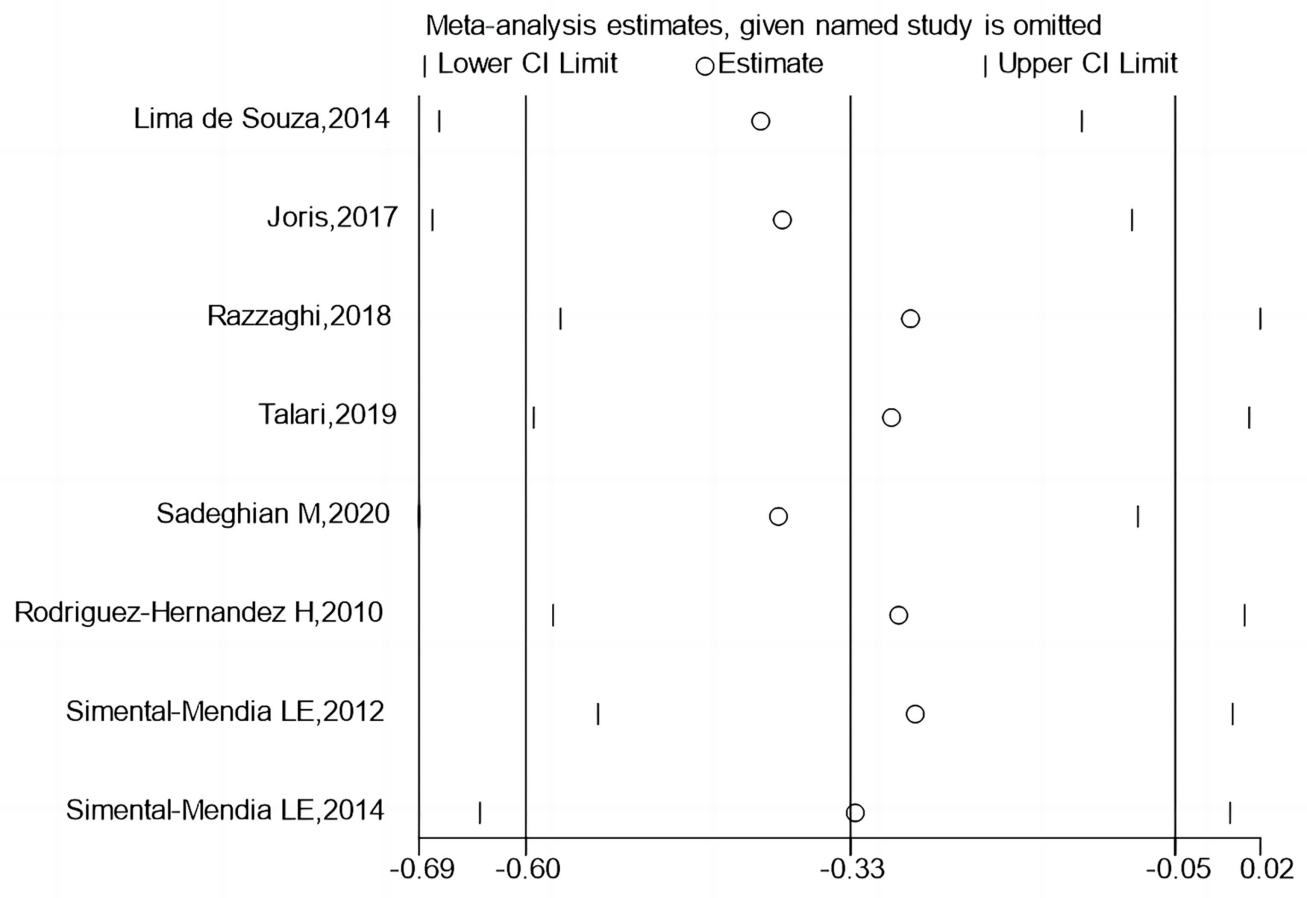
Sensitivity analysis by excluding each study one by one.

Figures 6, 7 report that sequential exclusion of each study from the meta-analysis model did not significantly alter the pooled effect size, with all results remaining statistically significant.

### 3.7 Publication bias

**Figure 8:**
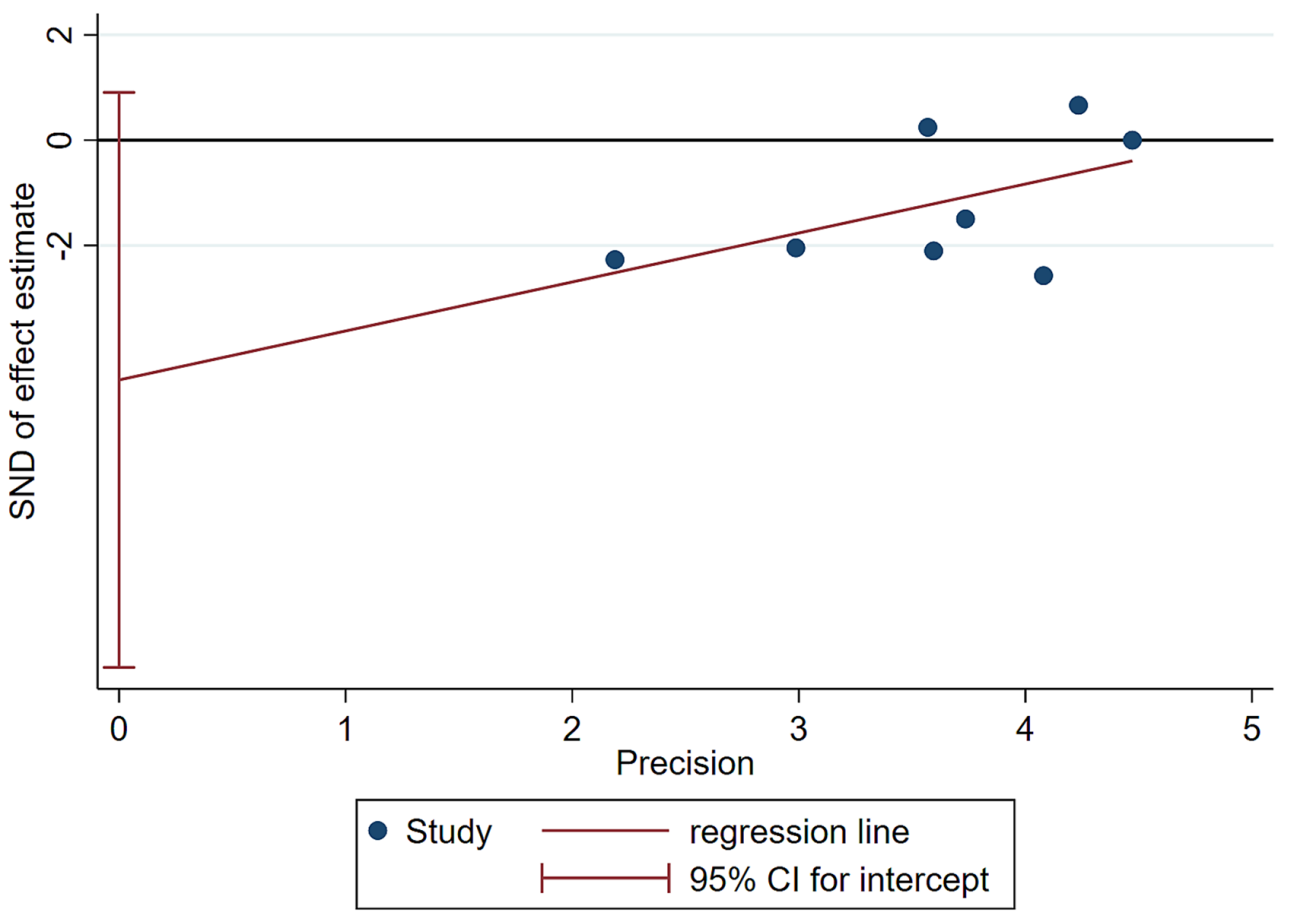
Sensitivity analysis by excluding each study one by one.

**Figure 9:**
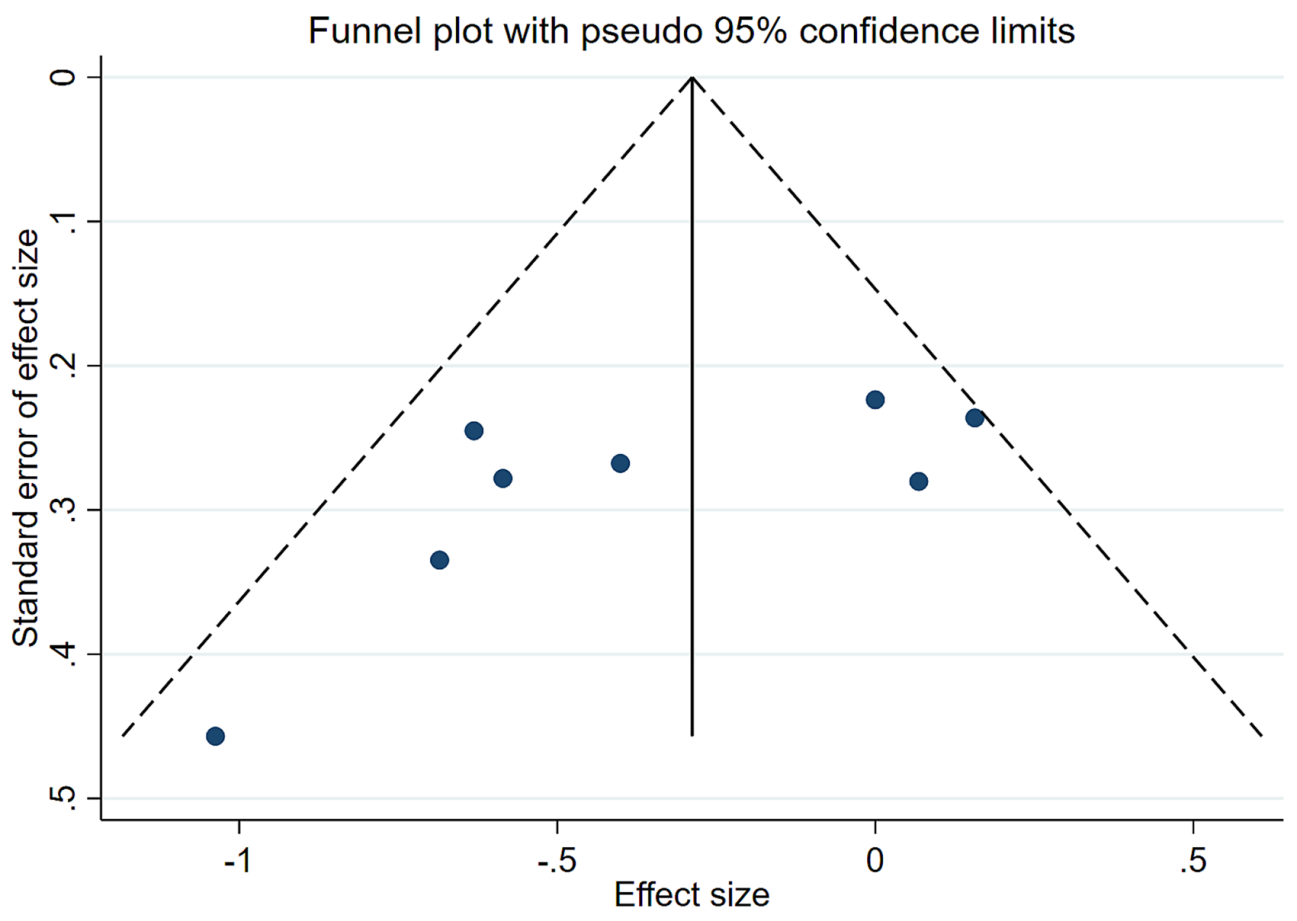
Funnel plot for publication bias.

The funnel plot in Figure 8 demonstrated symmetry (*p*-values of 0.1078 for Begg’s test and 0.087 for Egger’s test, both *p* > 0.05), indicating no significant publication bias (Figure 9).

### 3.8 GRADE analysis

The quality of evidence regarding the effect of magnesium intake on CRP was upgraded to high, owing to serious inconsistency within the GRADE domains. For IL-6, the quality of evidence was rated moderate due to serious imprecision and serious inconsistency. In contrast, the evidence concerning the impact of magnesium intake on TNF-α was upgraded to high as a result of serious imprecision in the GRADE domains. The GRADE profile is shown in Table 5.

**Table 5:** GRADE profile of magnesium supplementation on serum inflammatory parameters.

Outcomes	Risk of bias	Inconsistency	Indirectness	Imprecision	Publication bias	Quality of evidence
CRP	No serious limitations	Serious limitations	No serious limitations	No serious limitations	No serious limitations	⊕ ⊕ ○ ○ Moderate
IL-6	No serious limitations	Serious limitations	No serious limitations	Serious limitations	No serious limitations	⊕ ⊕ ○ ○ Moderate
TNF-α	No serious limitations	No serious limitations	No serious limitations	Serious limitations	No serious limitations	⊕ ⊕ ○ ○ Moderate

## 4 Discussion

As far as we are aware, it represents the initial meta-analysis to explore the association. Between long-term magnesium consumption and inflammatory markers in patients with metabolic syndrome. Longitudinal magnesium consumption appears to offer prophylactic benefits for reducing C-reactive protein levels. Although the results indicated a reduction in glutathione levels, the limited number of studies (fewer than three) investigating glutathione precludes definitive conclusions regarding the impact of long-term magnesium consumption on glutathione levels. MetS, a multifaceted condition marked by abdominal obesity, impaired glucose metabolism, high blood pressure, and abnormal lipid levels, is pathophysiologically linked to chronic low-grade inflammation. Inflammation both results from and accelerates metabolic syndrome (MetS) onset and advancement (24). Magnesium (Mg), the human body’s fourth most prevalent crucial mineral, has recently been recognized for its important roles in modulating inflammatory responses and improving metabolic abnormalities. Numerous studies (25–27) have demonstrated that patients with MetS commonly exhibit reduced serum magnesium levels, and this magnesium-deficient state may exacerbate inflammatory responses and promote metabolic dysregulation. Therefore, investigating the effects of magnesium intake on inflammatory markers in MetS patients holds significant clinical relevance.

From a pathophysiological perspective, dysregulated immune responses are central to metabolic syndrome etiology and advancement. Excessive buildup of abdominal fat causes adipocyte hypertrophy and local hypoxia, which subsequently activate the innate immune system and promote macrophage polarization toward the pro-inflammatory M1 phenotype. These activated immune cells secrete abundant pro-inflammatory cytokines, including TNF-α, IL-6, and MCP-1, while suppressing anti-inflammatory factors such as adiponectin. This pro-inflammatory microenvironment interferes with insulin signaling through multiple molecular mechanisms, including increased serine phosphorylation and decreased tyrosine phosphorylation of insulin receptor substrate-1 (IRS-1), thereby inhibiting normal activation of the phosphatidylinositol 3-kinase (PI3K)/protein kinase B (Akt) signaling pathway, ultimately leading to the development of insulin resistance (28, 29). Insulin resistance further exacerbates glucose and lipid metabolic disorders, forming a vicious cycle. Additionally, chronic inflammation exacerbates cardiovascular complications, such as atherosclerosis and hypertension, through mechanisms including vascular endothelial cell activation, oxidative stress promotion, and vascular smooth muscle dysfunction. Hyperglycemia and elevated free fatty acids (FFA) can reciprocally activate the NLRP3 inflammasome, establishing a “metabolism-inflammation” positive feedback loop (30). Gut microbiota dysbiosis (e.g., altered Firmicutes/Bacteroidetes ratio) increases endotoxin (LPS) translocation into circulation, triggering immune responses via Toll-like receptor 4 (TLR4), promoting systemic low-grade inflammation, and affecting bile acid metabolism and short-chain fatty acid (SCFA) production, thereby further exacerbating metabolic abnormalities in MetS (31).

Magnesium participates in over 600 enzymatic reactions in the human body, playing indispensable roles in physiological processes including energy metabolism, nucleic acid and protein synthesis, ion channel regulation, and cellular signal transduction (32). Epidemiological studies (33) have demonstrated that serum magnesium levels in metabolic syndrome patients are generally lower than in healthy populations. This magnesium-deficient state may be associated with multiple factors, including insufficient magnesium intake due to modern refined diets, increased renal magnesium excretion induced by insulin resistance, and magnesium metabolism disorders caused by chronic inflammation. The mechanisms by which magnesium deficiency affects metabolic syndrome are complex: in glucose metabolism, magnesium serves as a cofactor for key glycolytic and gluconeogenic enzymes, and its deficiency directly impairs glucose metabolism (34); magnesium ions also regulate insulin receptor affinity and tyrosine kinase activity (12, 35), with deficiency leading to impaired insulin signaling. In lipid metabolism, magnesium deficiency reduces lipoprotein lipase activity, impairs very-low-density lipoprotein (VLDL) clearance and promotes free fatty acid release (23). Regarding vascular function, magnesium deficiency decreases nitric oxide (NO) bioavailability, stimulates endothelin-1 secretion, and enhances vascular sensitivity to angiotensin II, consequently promoting vasoconstriction and elevated blood pressure (36). Increasing magnesium intake through diet or supplements can significantly improve various metabolic abnormalities in metabolic syndrome patients, including enhanced insulin sensitivity, healthier glucose control (fasting & after meals), better lipid readings, and regulated blood pressure. These beneficial effects are not only attributable to magnesium’s direct involvement in metabolic regulation but may also partially stem from its anti-inflammatory properties. Magnesium’s anti-inflammatory mechanisms operate at three levels: molecularly, magnesium curtails NF-κB cascade initiation (37), whereas magnesium deficiency promotes NF-κB nuclear translocation, increasing transcription and secretion of inflammatory cytokines, including TNF-α and IL-6; at the organelle level, magnesium regulates NOD-like receptor thermal protein domain-associated protein 3 (NLRP3) inflammasome assembly and activation. By maintaining intracellular ion balance and mitochondrial function, magnesium reduces reactive oxygen species (ROS) production, thereby suppressing NLRP3 inflammasome activation and preventing the generation of IL-1β and IL-18 (37); systemically, as an essential cofactor for antioxidant enzymes (38), magnesium alleviates oxidative stress-mediated inflammatory responses while also modulating hypothalamic–pituitary–adrenal axis function to influence glucocorticoid secretion and systemic inflammatory regulation.

The current research findings still exhibit some inconsistencies, with certain randomized controlled trials failing to demonstrate significant improvements in inflammatory markers after magnesium supplementation. These discrepancies may be attributed to multiple factors, including baseline characteristics of study participants (e.g., age, sex, degree of obesity, and baseline magnesium status), dosage and form of magnesium supplements (organic magnesium salts generally exhibit better bioavailability than inorganic salts), intervention duration (short-term interventions may be insufficient to observe notable anti-inflammatory effects), and variations in inflammatory marker assessment methodologies. Additionally, interactions between magnesium and other nutrients may influence its anti-inflammatory efficacy; for instance, vitamin D enhances intestinal magnesium absorption, while zinc and calcium may compete with magnesium for absorption—all these factors require careful consideration when designing studies and interpreting results. From a clinical application perspective, although magnesium supplementation shows promising potential, practical interventions should adhere to personalized approaches, as metabolic syndrome patients with different magnesium nutritional statuses may require distinct intervention strategies. Concurrently, the safe intake range of magnesium should be considered to avoid adverse effects, such as diarrhea and nausea, caused by excessive intake. More importantly, obtaining sufficient magnesium through a balanced diet may offer more comprehensive health benefits than magnesium supplements alone. Sources of Magnesium: unrefined cereals, verdant greens, nuts, legumes, and seafood, which provide not only magnesium but also other health-promoting nutrients and phytochemicals. Future research should focus on the following aspects: first, conducting larger-scale, long-term follow-up randomized controlled trials to evaluate the long-term effects of different magnesium supplementation doses and forms on inflammatory status and clinical hard endpoints in patients with metabolic syndrome; second, investigating the synergistic mechanisms between magnesium and other nutrients in regulating inflammatory responses to provide a theoretical basis for developing combined intervention strategies; third, exploring individual variations in magnesium’s anti-inflammatory effects and their molecular foundations to inform precision nutrition interventions; and finally, developing more accurate and convenient methods for assessing magnesium nutritional status to better guide clinical practice.

C-reactive protein (CRP) is widely recognized as one of the most reliable and direct markers of inflammation. It is synthesized by the liver in response to pro-inflammatory cytokines, particularly interleukin-6 (IL-6), and serves as a key indicator of systemic inflammation. CRP has been consistently shown to correlate with a variety of inflammatory conditions, including metabolic syndrome, cardiovascular disease, and diabetes, making it a valuable biomarker for clinical practice. In comparison to other inflammatory markers such as IL-6 and TNF-α, CRP has the advantage of being more readily available and less variable, which makes it particularly suitable for large-scale epidemiological studies and clinical monitoring. While IL-6 and TNF-α are also important markers of inflammation, they are often more difficult to measure consistently across studies due to variability in assay techniques and the presence of confounding factors. Therefore, CRP was chosen as the primary marker in our analysis, as it is not only the most frequently reported but also provides a clear and direct reflection of inflammatory status in individuals with metabolic syndrome. Given the scarcity of data on other biomarkers, including IL-6 and TNF-α, we focused on CRP to provide a more robust and clinically meaningful evaluation of the effects of magnesium supplementation. Future studies with larger sample sizes and more consistent reporting of additional biomarkers may allow for a more comprehensive analysis of the full spectrum of inflammatory responses associated with metabolic syndrome.

Several potential confounders could influence the outcomes of magnesium supplementation in individuals with metabolic syndrome. Dietary intake is an important factor, as the baseline magnesium levels in participants could vary depending on their diet, potentially affecting the response to supplementation. For instance, individuals with a magnesium-rich diet might experience less pronounced effects from supplementation compared to those with a magnesium-deficient diet. Comorbidities such as diabetes, hypertension, and obesity are also critical confounders, as these conditions can independently affect inflammatory markers and metabolic regulation. Additionally, the use of medications (e.g., statins, antihypertensives, or insulin) may influence both the inflammatory process and magnesium metabolism. Unfortunately, most studies included in this review did not report sufficient data on these variables, preventing us from adjusting for them in our analysis. Future studies should consider these factors and aim to report detailed information on baseline dietary intake, comorbid conditions, and medication use to better isolate the effects of magnesium supplementation and provide a more comprehensive understanding of its role in managing metabolic syndrome.

Furthermore, our meta-analysis has several limitations that should be considered. First, the majority of the included studies (seven out of eight) were conducted in developing countries. This may limit the generalizability of our findings to developed nations, where healthcare systems, socioeconomic status, and dietary habits may differ substantially. Future studies should include more data from developed countries to validate and extend our conclusions. This analysis has limitations. First, although we focused on metabolic syndrome, not all included studies used formal MetS criteria. We adopted a pragmatic approach by including studies of patients with core MetS components (e.g., diabetes, obesity, dyslipidemia, hypertension) per standard definitions (39, 40), which may introduce population heterogeneity. Second, observed heterogeneity may stem from variations in magnesium dosage, intervention duration, and participant baselines. Finally, the small number of studies for specific inflammatory markers limits generalizability.

In conclusion, from a clinical perspective, these findings suggest that magnesium supplementation may be considered as an adjunctive strategy for managing inflammation in individuals with metabolic syndrome. Based on the available evidence, oral supplementation at typical doses of 250–400 mg elemental magnesium per day for 12 to 16 weeks appears to be both effective and generally well tolerated. However, clinicians should exercise caution when recommending magnesium to patients with renal impairment or those taking multiple medications, as reduced renal clearance may increase the risk of hypermagnesemia and drug interactions. Further large-scale trials are warranted to confirm optimal dosing regimens and evaluate long-term safety across diverse patient populations. Current evidence suggests that magnesium deficiency may exacerbate low-grade inflammation and thereby promote the onset and advancement of metabolic syndrome. Appropriate magnesium supplementation shows promise as a potential intervention strategy for improving inflammatory status and metabolic abnormalities in patients with metabolic syndrome. However, higher-quality studies are needed to optimize intervention protocols and clarify long-term benefits. In clinical practice, magnesium supplementation strategies should be incorporated into a comprehensive lifestyle intervention framework, integrating dietary modifications, physical exercise, and behavioral changes to provide more holistic and effective solutions for mitigating and treating metabolic syndrome.

## 5 Conclusion

This systematic review and meta-analysis confirms prolonged magnesium intake substantially lowers C-reactive protein in individuals with metabolic syndrome, but does not reinforce the positive impact of magnesium intake on additional serum inflammatory indicators including IL-6, TNF-α, NO, MDA, TAC, and GSH in this population. However, the study has limitations; the currently available clinical data remain insufficient to guide clinical practice, and further high-quality controlled clinical trials are warranted to validate these findings.

## Data Availability

The raw data supporting the conclusions of this article will be made available by the authors, without undue reservation.

## Footnotes

^1^ http://www.ncbi.nlm.nih.gov/pubmed

^2^ https://www.scopus.com

^3^ http://www.webofscience.com
