# Supplementary material for: The effect of long-term magnesium intake on inflammatory markers in patients with metabolic syndrome: a systematic review and meta-analysis of randomized controlled trials
Source: Front Nutr. 2025 Oct 31;12:1692937. doi: 10.3389/fnut.2025.1692937 (PMC12617300; doi:10.3389/fnut.2025.1692937)
Supplement: Supplementary file 1 [file Image_1.pdf]

SUPPLEMENTARY

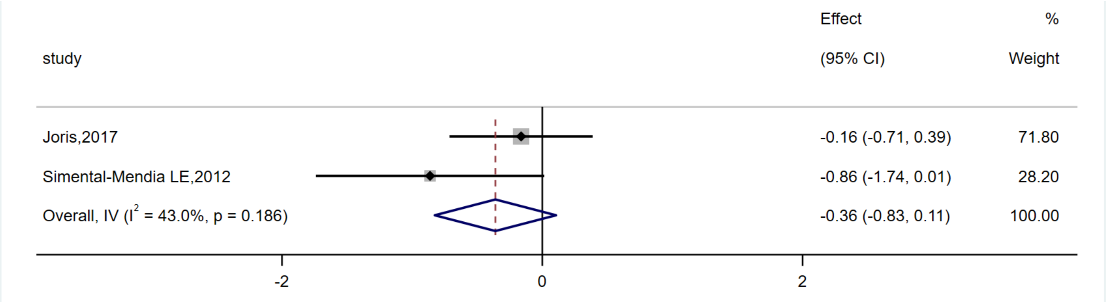

Figure 1.Forrest plot of the effect of magnesium versus placebo on serum interleukin-6.

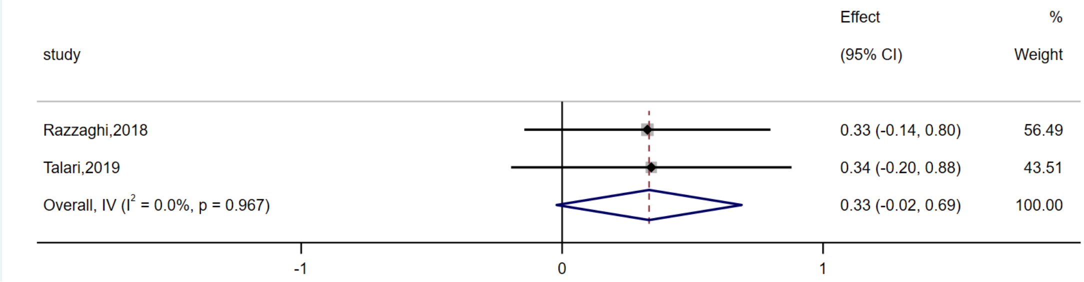

Figure 2.Forrest plot of the effect of magnesium versus placebo on serum nitric oxide.

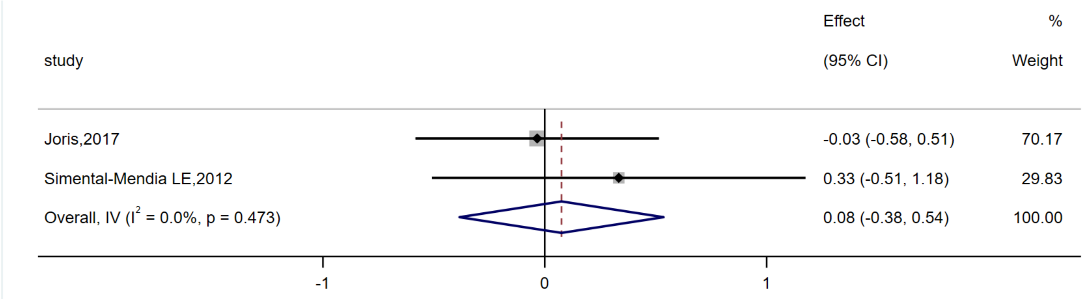

Figure 3.Forrest plot of the effect of magnesium versus placebo on serum tumor necrosis factor-alpha.

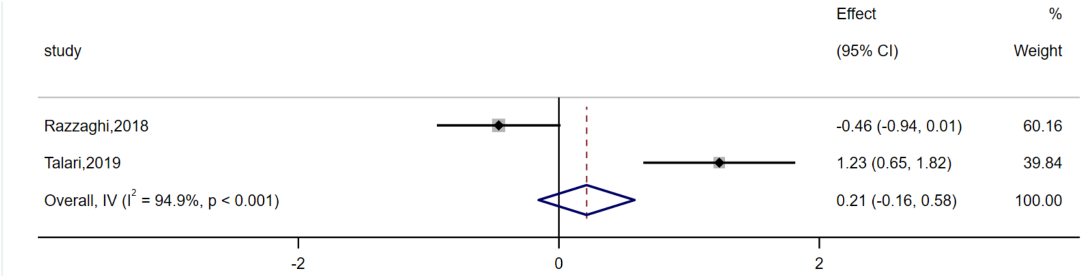

Figure 4.Forrest plot of the effect of magnesium versus placebo on serum total antioxidant capacity.

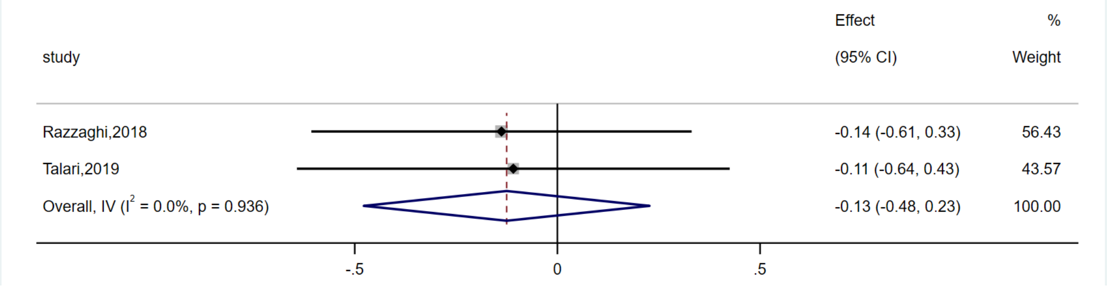

Figure 5.Forrest plot of the effect of magnesium versus placebo on serum glutathione.

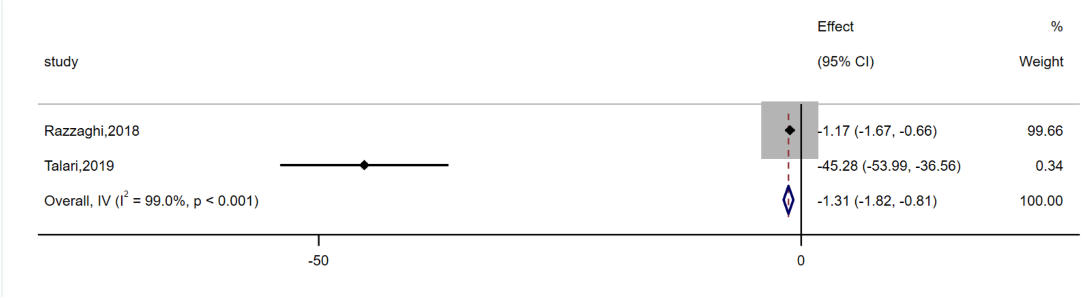

Figure 6.Forrest plot of the effect of magnesium versus placebo on serum malondialdehyde.
